# Realization of all two-dimensional Bravais lattices with metasurface-based interference lithography

**DOI:** 10.1515/nanoph-2023-0786

**Published:** 2024-01-15

**Authors:** Myungjoon Kim, Nayoung Kim, Jonghwa Shin

**Affiliations:** KAIST, Daejeon, Republic of Korea

**Keywords:** metasurface, interference lithography, proximity-field nanopatterning, adjoint method

## Abstract

Proximity-field nanopatterning (PnP) have been used recently as a rapid, cost-effective, and large-scale fabrication method utilizing volumetric interference patterns generated by conformal phase masks. Despite the effectiveness of PnP processes, their design diversity has not been thoroughly explored. Here, we demonstrate that the possibility of generating any two-dimensional lattice with diverse motifs. By controlling the amplitude, phase, and polarization of each diffraction beam, we can implement all two-dimensional Bravais lattices in three-dimensional space. The results may provide diverse applications that require three-dimensional nanostructures from optical materials and structural materials to energy storage or conversion materials.

## 1 Introduction

Three-dimensional nano- and micro-scale structures have been actively studied in various fields, including photonic crystals for band-gap engineering [1]–[3], mechanical metamaterials [4]–[7], electrochemical platforms [8], and electronic devices [9]. Their properties are significantly influenced by the regular arrangement of nanostructures, thereby increasing the importance of the fabricating periodic nanoscale patterns on a large scale. Among various fabrication methods, such as direct laser writing [10], self-assembly methods [11], multiphoton lithography [12], [13], etc., interference lithography (IL) or holographic lithography has garnered significant attention owing to its ability for fast, cost-effective, and large area production of periodic structures via single or multiple exposures of multiple coherent beams [14]–[22].

IL forms the structure by recording the interference pattern in a photoresist by combining multiple beams. Depending on the wavevectors, intensities, phases, and polarizations of the beams, a wide variety of structures can be formed [23]. Popular examples include diamond structures in a face-centered lattice and woodpile and gyroid structures in a base-centered lattice [24], [25]. There are several different approaches generating such beams. A conventional approach is to utilize beam splitters, phase retarders, and other discrete optical elements to generate and control multiple beams. Alternatively, a spatial light modulator (SLM) can be employed, which allows the dynamic control of beams [26]–[28]. Both methods rely on free-space optics, which provide effective ways to manipulate multiple beams in well-controlled lab environments, but pose challenges in terms of the large-area applicability and stability of the setups with respect to thermal drifts, vibrations, or other environmental factors.

Proximity-field nanopatterning (PnP), utilizing a single beam and a phase mask, has been suggested as a potential solution [29]–[33]. In its simplest form, a PnP method employs binary gratings to generate multiple diffraction orders from a single incident beam. The choice of the width, height, and period of the grating affects the resulting interference patterns. Because the grating is in direct contact with the photoresist (with an optional thin spacer), the method is inherently robust against mechanical vibrations and drifts. In addition, it is relatively straightforward to adapt the method to a large surface. To obtain more diverse patterns, generalized gratings or metasurfaces can be utilized. Recently, it has been shown that the inverse design of metasurfaces significantly enlarges the range of realizable nanopatterns [34], [35]. For example, previously elusive rectangular lattice has been successfully fabricated using the optimized metasurface [35]. To date, it remains an academically interesting and practically important question how diverse lattices and motifs can be realized with this method.

Here, we prove that three-dimensional patterns with any two-dimensional Bravais lattice can be constructed with metasurface-based PnP methods. Moreover, it is demonstrated that diverse range of motifs can be formed using the method. The key enabling factor for such expansion of available geometries is the ability to manipulate individual phases of four diffraction orders in addition to their intensities, in contrast to previous PnP approaches that primarily focused on intensity control in three beam interference [35]. We explain our method in three parts. First, we illustrate how proper diffraction orders are selected to form the fundamental lattice vectors in the reciprocal domain of a target lattice type. Second, possible types of motifs are numerically explored with various combinations of diffraction intensities, phases, and polarization combinations. Finally, we utilize an adjoint-based inverse design method to discover nontrivial phase mask patterns for each of the five two-dimensional Bravais lattices. We present a set of proof-of-concept examples that were unattainable using the prior design approach. This generalized patterning method is expected to simplify the production of a range of devices, including nano- or micro-fluidic channels and optical, acoustic, and mechanical metamaterials.

## 2 Methods

### 2.1 Proximity-field nanopatterning

The PnP process is uniquely characterized by its use of elastomeric phase masks that make conformal contact with the surface of the photoresist film. In the patterning process, a collimated laser beam passes through the periodic mask, resulting in the generation of multiple diffracted beams that create a spatial intensity distribution. This distribution triggers photo-polymerization or cross-linking reactions inside negative-type photoresists, thereby rendering them insoluble in the high intensity regions of the interference pattern. (For positive resists, the high intensity regions become soluble, resulting in an inversed pattern.) Following the development process, the interference pattern is translated into the nanopattern, retaining only the insoluble regions of the photoresist.

**Figure 1:**
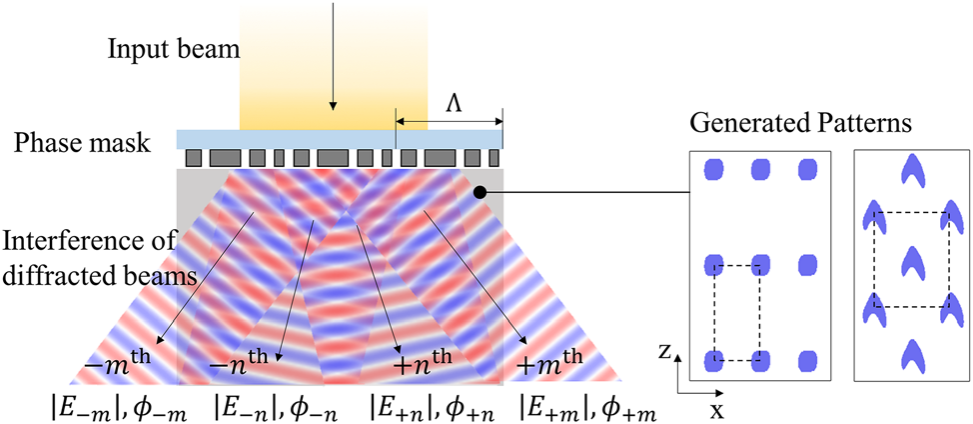
Schematic of proximity-field nanopatterning.

One can predict the resulting pattern by examining the multiple diffraction beams emanating from the phase mask. We consider phase masks that are periodic in the *x*-direction with period Λ and uniform in *y*-direction (Figure 1). Due to this symmetry, a single uniform plane wave normally incident on the mask will be scattered into a finite number of diffracted waves whose wavenumbers in the *x*-direction are integer multiples of 2π/Λ. While the electric field is a vector quantity, below, we use a scalar representation assuming a linear polarization that is either in the *x*- or *y*-direction, such that polarization is preserved after scattering due to the symmetry of the problem. In a general case in which the incident polarization is arbitrary, the resulting intensity pattern is a simple sum of individual intensity patterns of the *x*- and *y*-polarized cases. The chemical reaction is sensitive to the intensity of the total field, which can be expressed as

(1) $$I\left(r\right)=\underset{i}{\sum }E_{i}^{2}+\underset{i<j}{\sum }E_{i}E_{j}\cos\left(K_{i,j}\cdot r+\varphi_{i,j}\right),$$

where **r** represents the position vector, *E*_*i*_ corresponds to the electric field amplitude of the *i*th beam, and **K**_*i*,*j*_ = **k**_*i*_ − **k**_*j*_ and *φ*_*i*,*j*_ = *φ*_*i*_ − *φ*_*j*_ represent the differences between the wavevectors and phases of *i*th and *j*th beams, respectively. Because the mask is uniform in *y*, the wavevectors have only non-zero *k*_*x*_ and *k*_*z*_ components with $k_{mx}=m\cdot \Delta k=m\cdot \frac{2\pi }{\Lambda },\;m=0,\pm 1,\pm 2,\ldots$ . The expected pattern appearing on the negative photoresist after development is determined using a simple thresholding model [36]:

(2) $$P\left(r\right)=\left\{\begin{array}{cc} 1\; & \;\text{for }I\left(r\right)\geq I_{\text{th}},\\ 0\; & \;\text{for }I\left(r\right)<I_{\text{th}}, \end{array}\right.$$

where a value of 1 indicates that the material remains, while a value of 0 denotes that the material has been discarded after development, and *I*th is the threshold intensity. In practical fabrication process, *I*th can be controlled by changing the exposure dose and development conditions.

### 2.2 Two-dimensional Bravais lattices

Free-space IL with precisely controlled four non-coplanar beams can generate all fourteen three-dimensional (3D) Bravais lattices [16]. Likewise, it is straight-forward to show free-space IL with three non-collinear beams can produce all five 2D Bravais lattices. However, PnP systems are more limited than the free-space approach in which all diffracted beams propagate in inward directions, which implies that the inner product of their wavevectors with an outward surface-normal vector is always negative. Hence, the available configurations of the angles between different beams are constrained and no exactly opposing beams can be utilized. Nevertheless, the PnP system can also achieve all 2D Bravais lattices with proper selections of a specific set of diffraction orders tailored to the target lattice type as will be shown below.

**Figure 2:**
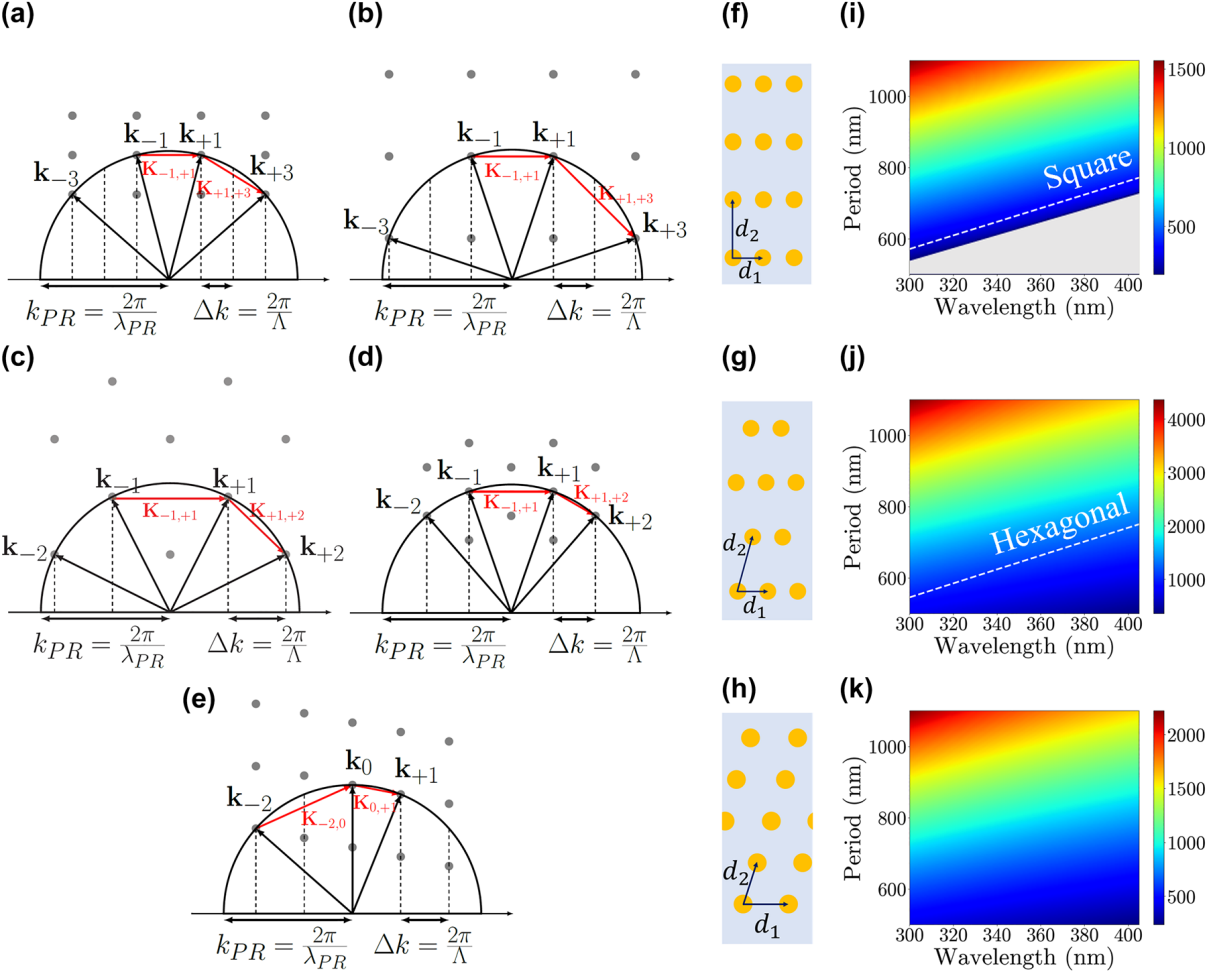
Selection of diffraction for two-dimensional Bravais lattices and the relationship between wavelength and mask period in the lattice constants. Lattice formation by diffraction orders is shown through equi-frequency contours and reciprocal lattice points. (a) Rectangular, (b) square, (c) centered rectangular, (d) hexagonal, and (e) oblique lattices are formed by selecting three diffraction beams. Primitive vectors are represented by red arrows. Lattice constants *d*_1_ and *d*_2_ illustrated for (f) rectangular and square, (g) centered rectangular and hexagonal, and (h) oblique lattices. The lengths of *d*_2_ for (h) rectangular and square, (i) centered rectangular and hexagonal, and (j) oblique lattice with respect to wavelength and period are represented as color maps.

In 2D, a Bravais lattice is uniquely determined by two basis vectors in the reciprocal space. As expressed in Eq. (1), the basis vectors are formed by the differences between the wavevectors of different diffraction orders. One can intuitively select a suitable set of diffraction orders for the target lattice by drawing the reciprocal lattice and equi-frequency contours, as illustrated in Figure 2. The equi-frequency contours are a collection of wavevectors corresponding to allow propagating modes in the photoresist for the particular choice of light frequency. In local media with negligible spatial dispersion, the contour is a circle whose radius is 2π*n*/*λ*, where *n* is the refractive index of the resist and *λ* is the vacuum wavelength of the illumination. This could be a non-trivial function of *λ* because *n* is also wavelength dependent for typical photoresists. In Figure 2, three diffraction orders required to form each lattice are represented as black arrows, and the reciprocal lattice points are depicted as gray dots. Note that except the oblique system, one can optionally utilize one more diffraction order (−3 for rectangular and square lattices, and −2 for centered rectangular and hexagonal lattices, respectively) to construct more diverse range of motifs as will be discussed in more details in Section 2.3. Based on the relation between real and reciprocal lattices, one can determine the lattice points in reciprocal space from the desired lattice type and lattice constants in real space. The *x*-directional component of the first-order wave vectors, denoted as Δ*k*, determines the proper value of the mask period as Λ = 2π/Δ*k*. Also, since any three distinct points on a plane uniquely determines the circle that passes through all three of them, the equi-frequency contour is fixed. Hence, from the radius of the equi-frequency contour, one can find the proper illumination wavelength. In this work, we choose SU-8 as the photoresist, whose refractive index varies between 1.68 and 1.65 in the wavelength range from 300 nm to 405 nm.

#### 2.2.1 Rectangular and square lattices

A rectangular lattice is formed when the two basis vectors are orthogonal to each other. However, there are no two points on a semi-circular equi-frequency contour that are vertically displaced from each other (One of the vertically opposing points would lie on the other semi-circle that corresponds to waves propagating upwards). Hence, instead of directly implementing two orthogonal vectors, one can realize one horizontal basis vector and one slanted vector, which is the addition of the horizontal and vertical basis vectors. Then, they can be matched to **K**_−1,+1_ and **K**_+1,+3_, respectively. A third-order diffracted beam is selected because **K**_−1,+1_ and **K**_+1,+3_ have the same *x*-component of 4π/Λ (Figure 2(a)). If a second order is chosen, it would become a centered rectangular or hexagonal lattice, as will be explained shortly. The lattice constants of the resulting photoresist pattern (Figure 2(f)) are expressed as

(3) $$d_{1}=\Lambda /2$$

(4) $$d_{2}={\left(\sqrt{\lambda_{\text{PR}}^{-2}-\Lambda^{-2}}-\sqrt{\lambda_{\text{PR}}^{-2}-9\Lambda^{-2}}\right)}^{-1}$$

where *λ*_PR_ = *λ*/*n* is the wavelength inside the photoresist. The square lattice is the special case of rectangular lattice when *d*_1_ = *d*_2_, which happens when *x*- and *z*-components of **K**_+1,+3_ are the same in magnitude (Figure 2(b)). It can be observed that *d*_1_ (the lateral length between lattice points) is solely defined by the period of the metasurface. On the other hand, *d*_2_ (the vertical lattice constant) is determined by both the wavelength and the mask period, as shown in Figure 2(i). For example, if a rectangular lattice with *d*_1_ = 500 nm and *d*_2_ = 1000 nm is desired, Λ = 1000 nm and *λ* = 360 nm can be selected. Note that in some photoresists, the resist shrinks vertically upon illumination due to chemical reactions. The shrinking ratio is consistent under controlled environments and can be considered when choosing Λ and *λ* [32].

#### 2.2.2 Centered rectangular and hexagonal lattices

Next, the centered rectangular lattice can be constructed by selecting diffraction orders (−1, + 1, + 2) or (−2, + 2, + 4) (Figure 2(c)). Similar to the previous calculation, the lattice constants *d*_1_ and *d*_2_ in the spatial domain (Figure 2(g)) are expressed as

(5) $$d_{1}=\Lambda$$

(6) $$d_{2}=\frac{1}{2}\sqrt{\Lambda^{2}+{\left(\sqrt{\lambda_{\text{PR}}^{-2}-\Lambda^{-2}}-\sqrt{\lambda_{\text{PR}}^{-2}-4\Lambda^{-2}}\right)}^{-2}},$$

and the hexagonal lattice is a special case of centered rectangular lattices when *d*_1_ = *d*_2_ (Figure 2(d)). Again, the horizontal lattice constant *d*_1_ is solely determined by the mask period.

#### 2.2.3 Oblique

The remaining case is the oblique lattice with the least symmetric properties. Consequently, any of the three diffraction order that do not fall under the categories of previous systems result in oblique lattice. Here, we select (−2, 0, + 1) diffraction orders (Figure 2(e)). The lengths *d*_1_ and *d*_2_ of the pattern in real space (Figure 2(h)) can be expressed as

(7) $$d_{1}=\Lambda$$

(8) $$d_{2}=\sqrt{\frac{2\Lambda^{2}\left(1-\lambda_{\text{PR}}\sqrt{\lambda_{\text{PR}}^{-2}-\Lambda^{-2}}\right)}{{\left(3-\lambda_{\text{PR}}\left(\sqrt{\lambda_{\text{PR}}^{-2}-4\Lambda^{-2}}+2\sqrt{\lambda_{\text{PR}}^{-2}-\Lambda^{-2}}\right)\right)}^{2}}}$$

Similar to the previous cases, the selection of specific wavelengths and periods of the phase mask offers a broad range of lattice lengths and angles based on Eq. (7) and (8) (Figure 2(k)). The detailed relationship between diffraction orders and lattice vectors is described in the Supplementary Materials.

### 2.3 Diversity of possible motifs

**Figure 3:**
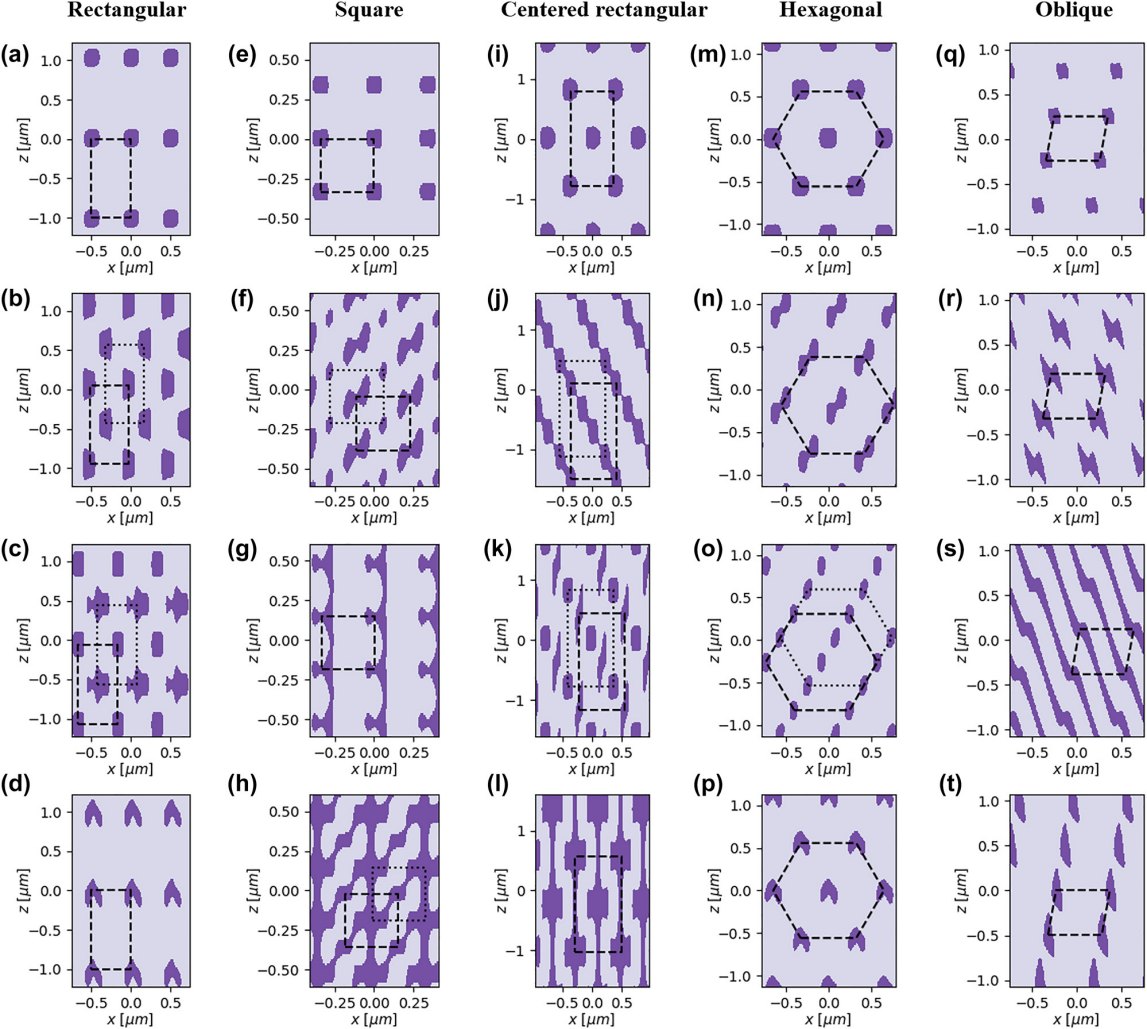
Diversity of the motifs on two-dimensional Bravais lattices. (a–d) Retangular, (e–h) square, (i–l) centered rectangular, (m–p) hexagonal, and (q–t) oblique lattice examples are shown. A single input beam is used to form (a–c, e–g, i–k, m–o, q), and the two beams with different polarization are used to create (d, h, l, p, r–t). The dark color denotes the regions lower than the threshold intensity. The dashed- and dotted-lines represent the lattices of each structure.

In addition to the Bravais lattice, creating desired motifs is crucial for nanopatterning. In the preceding section, we only considered three beams, the minimum required to create two-dimensional Bravais lattices. If the wavevectors of the three beams are **k**_1_, **k**_2_, and **k**_3_, the intensity maxima appear when

(9) $$K_{1,2}\cdot r+\varphi_{1,2}=2p\pi ,\;p=0,\pm 1,\pm 2,\ldots$$

(10) $$K_{1,3}\cdot r+\varphi_{1,3}=2q\pi ,\;q=0,\pm 1,\pm 2,\ldots$$

are satisfied [37]. When employing only these three beams, any change in their phase terms *φ*_1_, *φ*_2_, and *φ*_3_ solely results in simple horizontal and vertical shifts of the entire interference pattern, while the lattice and motifs remain unperturbed [15]. In a more general case with more than three coplanar beams, the relative phases become crucial and can be used to diversify the possible shapes of the motifs. If the wavevectors of the additional beams do not fall on one of the reciprocal lattice points, they break the periodicity of the original lattice and lead to the formation of quasi-periodic patterns. As we focus on achieving complex motifs while maintaining the periodicity of the desired lattice system, the possible choices of the additional diffraction orders are the other reciprocal lattice points coinciding with the semi-circular equi-frequency contour in Figure 2. This ensures that the lattice type and lattice constants remain unchanged. For all 2D Bravais lattices except the oblique lattice, there exists at least one more reciprocal lattice point guaranteed to be on the equi-frequency contour, namely, the negative third diffraction order for the rectangular and square lattices and the negative second for the centered rectangular and hexagonal lattices. So, one can utilize at least four beams for those lattices. By choosing certain combinations of the mask period and wavelength, additional diffraction orders can be brought to coincide with the equi-frequency contour, which is potentially useful for realizing even more diverse classes of motifs. Even with just four beams, adjusting the relative intensities and phases of each diffraction order produces various distinct motifs, as depicted in Figure 3. As a concrete example, we assumed a typical PnP configuration with a UV laser at 355 nm wavelength and a negative SU-8 photoresist with refractive index *n*_PR_ = 1.66 for these cases.

Unlike using an asymmetric three-beam configuration, the four-beam configuration is capable of producing a symmetric motif such as circles and rounded squares if the amplitude and phases are also symmetric (Figure 3(a, e, i, m)). Additionally, by adjusting the relative phases, one can make di-atomic motifs, where one component of the motif is offset from the other in both lateral and longitudinal directions, as depicted in Figure 3(b, c, f, j, k, o). While the “offset” rectangular crystals may resemble the centered rectangular crystals, and the “offset” centered rectangular crystals, the oblique crystals, respectively, an important distinction is that the offset crystals are di-atomic with potentially very different two sub-motifs.

To further enhance the control over diffracted beams and offer more degrees of freedom, a circular or elliptical polarization can be employed. Then, each diffraction order can have both *p*-polarized and s-polarized components. Since those two polarizations are orthogonal to each other, the total field intensity is the linear sum of the individual intensities from each polarization. The *p*-polarized beams contribute to the field intensity with $I_{\text{p}}={\left|E_{x}\right|}^{2}+{\left|E_{z}\right|}^{2}$ , while the *s*-polarized beams contribute to $I_{\text{s}}={\left|E_{y}\right|}^{2}$ . Consequently, the total field intensity is obtained by summing these intensities as *I*_tot_ = *I*_p_ + *I*_s_. This approach ensures a more extended manipulation of the multiple diffracted beams, allowing complex pattern generation, such as a V-shaped motif (Figure 3(d, p)) and interconnected patterns (Figure 3(h, l)). Even the oblique structures, obtained with three diffracted orders, can have complex patterns when utilizing two polarized inputs, as demonstrated in Figure 3(r–t). More diverse patterns attainable using PnP are given in Supplementary Materials.

## 3 Inverse design of metasurface masks

The actual mask that can produce the target crystal structure can be obtained with an inverse design method. Recently, it was proved that full control of the amplitude, phase, and polarization of light transmission is possible with a bi-layer metasurface [38]. We adopt a similar bi-layer configuration for our mask to ensure sufficient degrees of design freedom. The interference patterns are calculated using finite-difference time-domain simulations, assuming a frequency-tripled Nd:YAG laser source at a 355 nm wavelength. The periods are selected based on the calculations of the previous section. To approximate the actual PnP setup, the simulated structure comprises multiple layers, including a transparent substrate (*n*_s_ = 1.4), a metasurface layer composed of a high index material with a refractive index of *n*_h_ = 2.87, an elastic material (*n*_l_ = 1.4), and a negative photoresist (*n*_PR_ = 1.66), whose material parameters are chosen considering quartz, TiO_2_, PDMS, and SU-8, respectively. Moreover, interlayers are present between two mask layers and between the mask and the photoresist. We employ a high refractive index polymer (*n*_*p*_ = 1.66) [39] in these interlayers to support the same number of propagating diffraction orders in the photoresist. A similar bi-layer phase mask configuration for PnP was experimentally demonstrated recently [33]. We conduct a topology optimization algorithm to implement the target diffraction amplitudes and phases. The metasurface masks are divided into discrete pixels. The general objective function of the phase mask design problem in PnP can be defined with the errors in the complex diffraction coefficients:

(11) $$f_{\mathrm{obj}}=\underset{m}{\sum }w_{m}{\left\|t_{m}-{\hat{t}}_{m}\right\|}_{2}^{2},$$

where $t_{m}\left({\hat{t}}_{m}\right)$ represent *m*th relative complex diffraction amplitudes of the current (target) device, and *w*_*m*_ denotes a weight that represents the relative importance of *m*th diffraction order.

For faster optimizations, the adjoint method is used [40], [41]. It calculates the gradient of the objective function with respect to the design parameters with only two simulations, forward simulation with normal incident light and adjoint simulation with a backward propagating source of each diffraction order [35], [42]. Starting with a random initial geometry, a metasurface mask is iteratively modified to minimize the objective function using the gradient descent method. The design variables can have continuous value ranging from *n*_l_ to *n*_h_. Throughout the optimization, the binarization strength steadily increases, thereby resulting in the eventual binarization of the design into two materials comprising PDMS (low index material) and TiO_2_ (high index material). In addition, we incorporate auxilliary filters to generate patterns with a realistic minimum feature size  (see Supplementary Materials).

**Figure 4:**
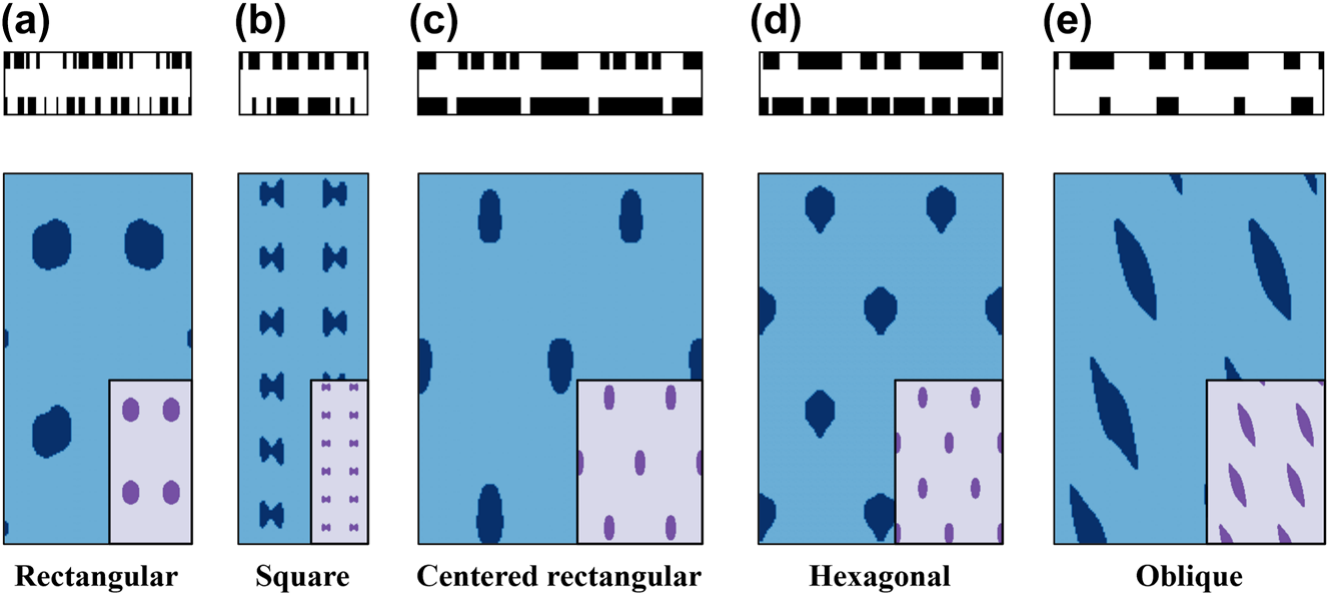
The optimized bi-layer mask designs, along with the resulting patterns (blue) and target patterns (purple). (a) A rectangular lattice with a 1000 nm period. (b) A square lattice with a 680 nm period. (c) A centered rectangular lattice with a 760 nm period. (d) A hexagonal lattice with a 650 nm period. (e) An oblique lattice with a 720 nm period. For (a–b), a single pattern period is displayed, while (c–e) present two periods to visualize lattice formation.

The optimization results of the five Bravais lattices with various motifs are illustrated in Figure 4. The patterns generated by optimized metasurface masks show good agreement with the target patterns. The slight irregularity in the patterns is owing to the non-zero amplitude of unwanted diffraction orders. According to the error analysis, it is observed that the requirement for the fabrication accuracy in mask shapes and the stability of laser intensity is not far from what can be achieved in a real experimental setting  (see Supplementary Materials). By decreasing the minimum feature size, the unwanted orders can be further minimized and more regular structures can be obtained.

## 4 Conclusions

We introduced a unified framework for metasurface-based interference lithography, allowing the implementation of a wide range of unit cell geometries. In particular, we demonstrated that all two-dimensional Bravais lattices can be realized using bi-layer masks. The optimal mask design can be obtained via adjoint-based optimizations. By manipulating the polarizations and phases, in addition to the intensities of diffraction orders, a diverse range of structures becomes available, and this approach can also be generalized for three-dimensional Bravais lattices, thus paving the way for advanced lithography techniques. While we focused on realizing complex motifs while preserving the periodicity in the target lattice system in this work, we note that a similar inverse design method utilizing the bi-layer metasurfaces could provide even more complex interference patterns if we relax our conditions on periodicity. This is an interesting research direction that can be potentially useful in generating periodic and non-periodic 3D structures (such as integrated photonic circuits) on the same platform.

## Supplementary Material

Supplementary Material Details
