# Supplementary material for: Realization of all two-dimensional Bravais lattices with metasurface-based interference lithography
Source: Nanophotonics. 2024 Jan 15;13(8):1467–74. doi: 10.1515/nanoph-2023-0786 (PMC11636505; doi:10.1515/nanoph-2023-0786)
Supplement: Supplementary file 1 — Supplementary Material Details [file j_nanoph-2023-0786_suppl_001.pdf]

Supplementary Materials: Realization of all two-dimensional Bravais lattices with metasurface-base interference

Myungjoon Kim, Nayoung Kim, and Jonghwa Shin
Department of Materials Science and Engineering,
KAIST, Daejeon, Republic of Korea

I. ADJOINT METHOD

Adjoint-based optimization has found applications in designing devices across various science and engineering disciplines, including aerodynamics[1,2], mechanical engineering[3], and photonics[4–7]. Notably, it has been used to create metasurfaces that can precisely control diffractions[6–8]. Here, we present an adjoint-based topology optimization approach for designing metasurface phase masks which induce predefined complex diffraction coefficients of multiple orders.

The simulation setting for the periodic metasurface is illustrated in Figure S1. The design variables $\rho = (\rho_1, \rho_2, ..., \rho_N)$ are set as continuous variables that range from 0 to 1 at each pixel $\mathbf{r}_i$ in the metasurfaces. The dielectric constant is given as

$$\epsilon_i = \rho_i \epsilon_{\mathrm{h}} + (1 - \rho_i)\epsilon_{\mathrm{l}}, \tag{1}$$

where ϵ_l and ϵ_h denote the low and the high index dielectric constants.

Complex diffraction coefficients are measured inside the photoresist. With the metasurface's period denoted as Λ, the complex diffraction coefficient for the mth order beam can be calculated as

$$t_m(\mathbf{E}, \mathbf{H}) = \int_{\mathbf{r}_0}^{\mathbf{r}_0+\Lambda} \left[\mathbf{E}(\mathbf{r}) \times \mathbf{H}_m^-(\mathbf{r}) - \mathbf{E}_m^-(\mathbf{r}) \times \mathbf{H}(\mathbf{r})\right] \cdot \mathbf{n}\, d\mathbf{r} \tag{2}$$

where $\mathbf{E}_m^-(\mathbf{r})$ and $\mathbf{H}_m^-(\mathbf{r})$ represent the normalized diffracted order field which propagate in backward direction.

In the main text, we define the objective function as

$$f_{obj} = \sum_m w_m \left\|t_m - \hat{t}_m\right\|_2^2 \tag{3}$$

where $\hat{t}_m$ represent m^{th} the desired complex diffraction coefficient. This objective functions have the value of 0 when the diffractions are completely controlled to be the target coefficients.

In order to obtain the graident of the objective function with respect to the design variables, we utilize the adjoint method. Suppose there is a small inclusion of dielectric perturbation $\Delta\epsilon$ at a specific point $\mathbf{r}'$, the change of the objective function owing to the inclusion can be represented as the overlap integral[6].

$$\Delta f_{obj} = 2\omega^2 \epsilon_0 \Delta\epsilon(\mathbf{r}') \Delta V \mathrm{Re}\left(\mathbf{E}(\mathbf{r}) \cdot \mathbf{E}^{\mathrm{adj}}(\mathbf{r})\right), \tag{4}$$

where the adjoint field $\mathbf{E}^{\mathrm{adj}}$ is defined as

$$\mathbf{E}^{\mathrm{adj}} = \sum_m w_m \left(\overline{t_m - \hat{t}_m}\right) \int \left[\mathbf{G}_{\mathrm{e}}(\mathbf{r}', \mathbf{r})\left(-\mathbf{n} \times \mathbf{H}_m^-(\mathbf{r})\right) - \mathbf{G}_{\mathrm{h}}(\mathbf{r}', \mathbf{r})\left(\mathbf{n} \times \mathbf{E}_m^-(\mathbf{r})\right)\right] \cdot \mathbf{n}\, d\mathbf{r} \tag{5}$$

where the overline indicates complex conjugation, and $\mathbf{G}_{\mathrm{e}}$, $\mathbf{G}_{\mathrm{h}}$ are the Green's tensors. Thus, the adjoint field is the sum of backward propagating beams with the angles specified by diffraction, and only two simulations are needed to calculate gradient of the design variables comprising phase masks.

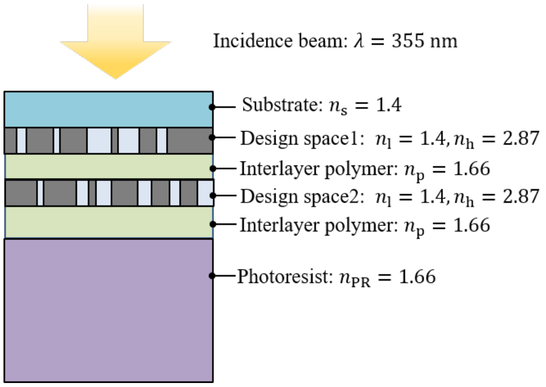

FIG. S1. Simulation setting for inverse design of phase mask.

To incorporate fabrication constraints, such as requirements for minimum grating feature sizes and binarization of materials, we used auxiliary filters in the topology optimization process. Initially, because of the constraints on the minimum line width that lithography can produce, the window averaging filter is applied to remove small features.

$$\tilde{\rho}_i = \frac{\sum_{j \in N_i} w_{ij}\rho_j}{\sum_{j \in N_i} \rho_j}, \quad w_{ij} = R - |r_i - r_j| \tag{6}$$

where R is the radius of the filter. Second, due to the necessity for the design spaces to incorporate two different materials - the phase mask material ($n_h = 2.87$) and the embedding material ($n_l = 1.40$) - a binary projection filter is utilized, which approximate step function through tanh function as

$$\bar{\rho}_i = \frac{\tanh \beta\eta + \tanh \beta(\tilde{\rho}_i - \eta)}{\tanh \beta\eta + \tanh \beta(1 - \eta)} \tag{7}$$

The hyperparameters β and η control the binarization strength and the threshold value, respectively. During the optimization, β varies by every 30 iterations, from 10 to 500.

Lastly, the gradient can be calculated using the chain rule:

$$\frac{\partial f_{\text{obj}}}{\partial \rho} = \frac{\partial f_{\text{obj}}}{\partial \epsilon} \cdot \frac{\partial \epsilon}{\partial \bar{\rho}} \cdot \frac{\partial \bar{\rho}}{\partial \tilde{\rho}} \cdot \frac{\partial \tilde{\rho}}{\partial \rho} \tag{8}$$

II. LATTICE FORMATION

Because the reciprocal lattice vectors $\mathbf{a}$ relate to the real lattice vectors $\mathbf{b}$ as $\mathbf{a} \cdot \mathbf{b} = 2\pi\delta_{ij}$, we can find the relation between pattern length and period of phase mask as Eqs.3–8 in the main text.

For the rectangular lattice, which can be formed by the selection of $(-1, +1, +3)$ diffraction orders, the reciprocal vectors are defined as

$$\mathbf{a}_1 = 2\Delta k_x \hat{x} \tag{9}$$

$$\mathbf{a}_2 = 2\Delta k_x \hat{x} - \Delta k_z^{\mathrm{r}} \hat{z} \tag{10}$$

where $\Delta k_x = 2\pi/\Lambda$ and $\Delta k_z^{\mathrm{r}} = \sqrt{k_{\mathrm{PR}}^2 - \Delta k_x^2} - \sqrt{k_{\mathrm{PR}}^2 - 9\Delta k_x^2}$ for simple notation.

The corresponding real lattice vectors can be calculated as

$$\mathbf{b}_1 = 2\pi \left(\frac{1}{2\Delta k_x} \hat{x} + \frac{1}{\Delta k_z^{\mathrm{r}}} \hat{z} \right) \tag{11}$$

$$\mathbf{b}_2 = 2\pi \left(-\frac{1}{\Delta k_z^{\mathrm{r}}} \hat{z} \right) \tag{12}$$

Therefore, the lengths of the rectangular pattern would be (Figure S2(a))

$$d_1 = \pi/\Delta k_x \tag{13}$$

$$d_2 = 2\pi/\Delta k_z^{\mathrm{r}} \tag{14}$$

The square lattice is uniquely identified when $\pi/\Delta k_x = 2\pi/\Delta k_z^{\mathrm{r}}$. In this case, the phase mask period is determined by a specific length with a fixed wavelength and refractive index of the photoresist.

For the centered-rectangular lattice, which can be formed by the selection of $(-1, +1, +2)$, the reciprocal vectors are defined as

$$\mathbf{a}_1 = 2\Delta k_x \hat{x} \tag{15}$$

$$\mathbf{a}_2 = \Delta k_x \hat{x} - \Delta k_z^{\mathrm{c}} \hat{z} \tag{16}$$

where $\Delta k_z^c = \sqrt{k_{\mathrm{PR}}^2 - \Delta k_x^2} - \sqrt{k_{\mathrm{PR}}^2 - 4\Delta k_x^2}$.

The corresponding real lattice vectors can be calculated as

$$\mathbf{b}_1 = \pi \left(\frac{1}{\Delta k_x} \hat{x} + \frac{1}{\Delta k_z^{\mathrm{c}}} \hat{z} \right) \tag{17}$$

$$\mathbf{b}_2 = 2\pi \left(-\frac{1}{\Delta k_z^{\mathrm{c}}} \hat{z} \right) \tag{18}$$

Therefore, the lengths of the centered-rectangular pattern would be (Figure S2(b))

$$d_1 = 2\pi/\Delta k_x \tag{19}$$

$$d_2 = \sqrt{(\pi/\Delta k_x)^2 + (\pi/\Delta k_z^{\mathrm{c}})^2} \tag{20}$$

The hexagonal lattice is uniquely identified when $\Delta k_x = \sqrt{3}\Delta k_z^{\mathrm{c}}$.

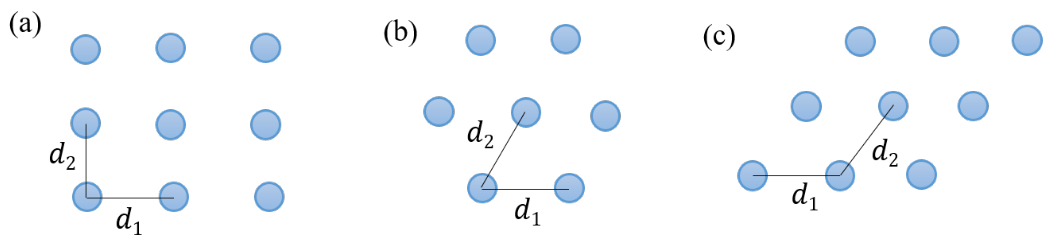

FIG. S2. Lattices in real space. (a) Rectangular (b) Centered-rectangular (c) Oblique.

For the oblique lattice, which can be formed by the selection of $(-2, 0, +1)$, the reciprocal vectors are defined as

$$\mathbf{a}_1 = \Delta k_x \hat{x} - \Delta k_{z1}^{\mathrm{o}} \hat{z} \tag{21}$$

$$\mathbf{a}_2 = -2\Delta k_x \hat{x} - \Delta k_{z2}^{\mathrm{o}} \hat{z} \tag{22}$$

where $\Delta k_{z1}^{\mathrm{o}} = k_{\mathrm{PR}} - \sqrt{k_{\mathrm{PR}}^2 - \Delta k_x^2}$ and $\Delta k_{z2}^{\mathrm{o}} = k_{\mathrm{PR}} - \sqrt{k_{\mathrm{PR}}^2 - 4\Delta k_x^2}$.

The corresponding real lattice vectors can be calculated as

$$\mathbf{b}_1 = 2\pi \left(\frac{\Delta k_{z2}^{\mathrm{o}}}{\Delta k_x \left(2\Delta k_{z1}^{\mathrm{o}} + \Delta k_{z2}^{\mathrm{o}}\right)} \hat{x} - \frac{2}{2\Delta k_{z1}^{\mathrm{o}} + \Delta k_{z2}^{\mathrm{o}}} \hat{z} \right) \tag{23}$$

$$\mathbf{b}_2 = -2\pi \left(\frac{\Delta k_{z1}^{\mathrm{o}}}{\Delta k_x \left(2\Delta k_{z1}^{\mathrm{o}} + \Delta k_{z2}^{\mathrm{o}}\right)} \hat{x} - \frac{1}{2\Delta k_{z1}^{\mathrm{o}} + \Delta k_{z2}^{\mathrm{o}}} \hat{z} \right) \tag{24}$$

The lengths of the oblique pattern can be calculated as (Figure S2(c))

$$d_1 = |b_{1,x}| + 2\,|b_{2,x}| = 2\pi/\Delta k_x \tag{25}$$

$$d_2 = |\mathbf{b}_2| = \sqrt{b_{2,x}^2 + b_{2,z}^2} \tag{26}$$

where $\mathbf{b}_l = b_{l,x}\hat{x} + b_{l,z}\hat{z}, \quad l = 1, 2.$

III. PATTERN LIBRARY

The intensity distribution results from the interference of multiple beam interference. Focusing only on instances where the diffracted beam propagates within $x - z$ plane, the total electric field can be represented as[9]

$$\mathbf{E}_{\mathrm{tot}} = \mathbf{E}_x \hat{x} + \mathbf{E}_y \hat{y} + \mathbf{E}_z \hat{z} \tag{27}$$

where

$$\mathbf{E}_x = \sum_m E_{m,p} \exp(i\mathbf{k}_m \cdot \mathbf{r} + i\varphi_{m,p}) \cos\theta_m \tag{28}$$

$$\mathbf{E}_y = \sum_m E_{m,s} \exp(i\mathbf{k}_m \cdot \mathbf{r} + i\varphi_{m,s}) \tag{29}$$

$$\mathbf{E}_z = \sum_m E_{m,p} \exp(i\mathbf{k}_m \cdot \mathbf{r} + i\varphi_{m,p}) \sin\theta_m \tag{30}$$

where the subscript p and s denote the polarizations, and θ_m is diffraction angles.

With the variation of the amplitude of electric fields (E_m), relative phase (φ_m), and the ratio of two polarized beams, a set of diverse patterns can be generated (Figure S3–S4).

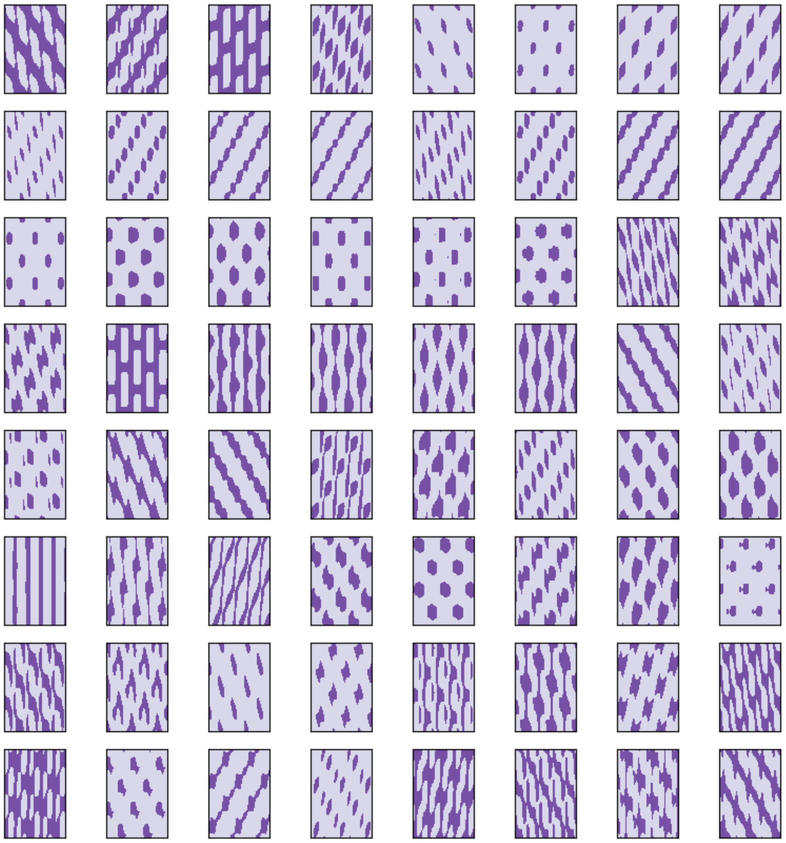

FIG. S3. Brute-force pattern generation for hexagonal lattice.

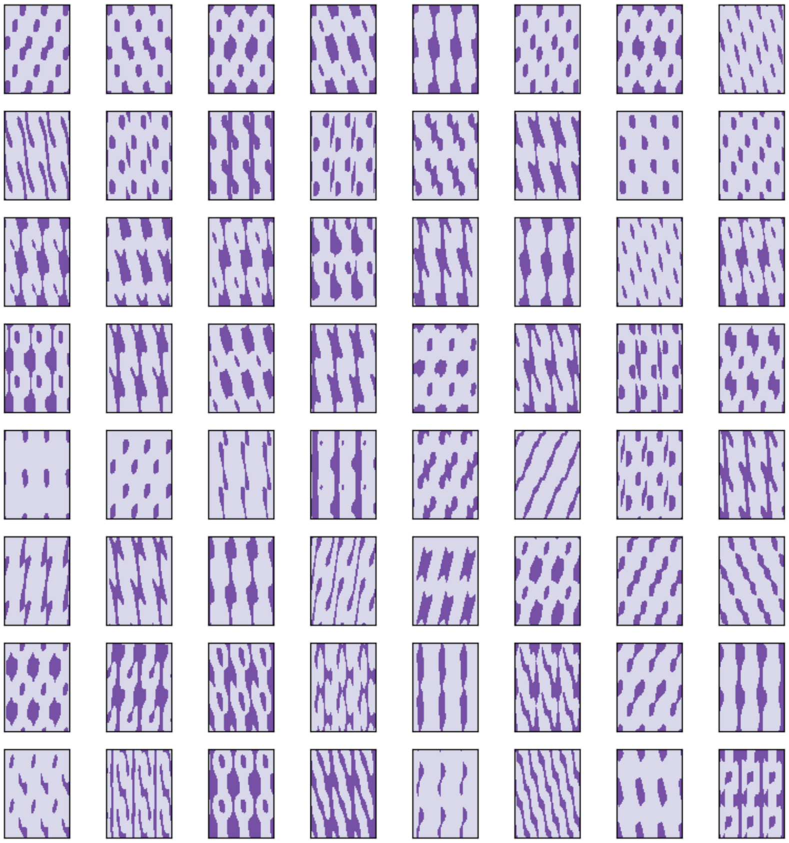

FIG. S4. Brute-force pattern generation for rectangular lattice.

IV. ERROR ANALYSIS

a. Fabrication error The fabrication errors may include disparities between the optimized and fabricated mask geometries, as well as misalignments in two-layer masks. These errors have the potential to impact the implementation of each diffraction efficiency, leading to shape deformations such as broken periodicity and non-uniform motifs in the final patterns. We conducted simulations to examine the influence of fabrication errors on the optimized structure of a centered rectangular lattice. In Figure S5 (a–b), the electric field intensity and corresponding structures of the original design are represented. Intentionally introducing 10 nm enlargement of the mask geometry results in patterns that are not significantly deformed, as shown in Figure S5 (d). On the other hand, 10 nm erosion results in somewhat noticeable changes S5 (c). Thus, in practice, the target mask geometry could be scaled to a slightly (e.g., 5 nm) larger size to maximize the fabrication error margin. Additionally, the pattern deformation due to misalignment, specifically 10 nm and 20 nm in two layers, is illustrated in Figure S5 (e–f). While fabrication errors in the mask have the potential to compromise the quality of the patterns, the requirements for fabrication accuracy is not far from what can be achieved in reality. To further enhance robustness to the fabrication error, we can adopt robust topology optimization method, which include the performances of deformed structures also into the objective functions[10]. This approach increases the tolerance towards fabrication errors to form desired patterns.

b. Intensity fluctuation We investigate the impact of laser source intensity fluctuations. As illustrated in Figure S5 (g–h), we varied the threshold intensity by 30 % above and below the original value. The resulting patterns indicate that the final pattern only undergoes a change in motif size while maintaining regularity. These results are due to the high contrast of intensity in the interference pattern. The commercially available laser sources used in the PnP process exhibit sufficient intensity stability, ensuring uniformity in the patterned structures. Moreover, the error-insensitive target interference mitigates the need for the precise control of laser intensity in the PnP process.

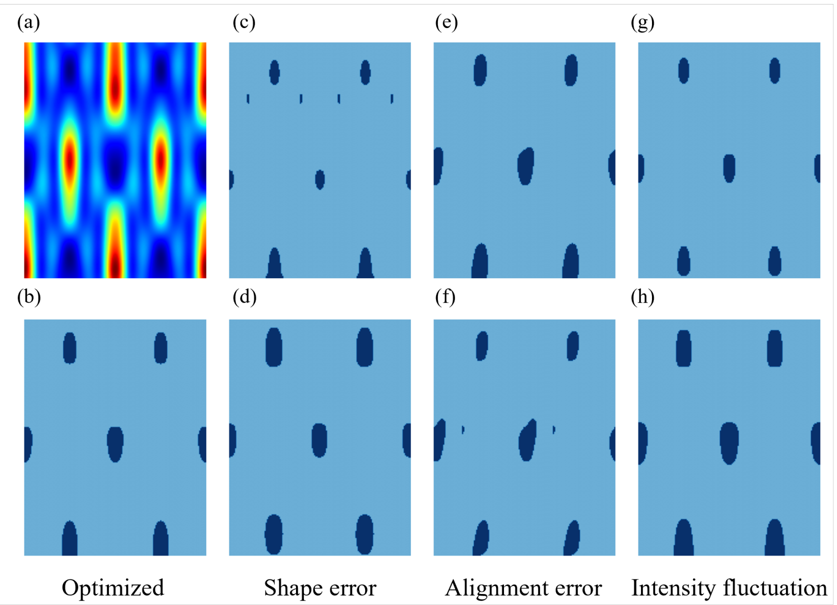

FIG. S5. Comparison of pattern formation under fabrication errors: (a, b) The interference of the optimized mask and the resulting pattern. Expected patterns generated by (c) eroded and (d) dilated masks, as well as misalignment of (e) 10 nm and (f) 20 nm in two-layer metasurface masks. The patterns generated by incident light with (g) 30% higher and (h) lower intensity than the original.

V. REFERENCE

[1]N. Aage, E. Andreassen, B. S. Lazarov, and O. Sigmund, "Giga-voxel computational morphogenesis for structural design," Nature **550**, 84–86 (2017).

[2]M. B. Giles and N. A. Pierce, "An introduction to the adjoint approach to design," Flow, turbulence and combustion **65**, 393–415 (2000).

[3]C. M. Pappalardo and D. Guida, "Adjoint-based optimization procedure for active vibration control of nonlinear mechanical systems," Journal of Dynamic Systems, Measurement, and Control **139**, 081010 (2017).

[4]H. Chung and O. D. Miller, "High-na achromatic metalenses by inverse design," Optics Express **28**, 6945–6965 (2020).

[5]H. Chung and O. D. Miller, "Tunable metasurface inverse design for 80% switching efficiencies and 144 angular deflection," Acs Photonics **7**, 2236–2243 (2020).

[6]D. Sell, J. Yang, S. Doshay, R. Yang, and J. A. Fan, "Large-angle, multifunctional metagratings based on freeform multimode geometries," Nano letters **17**, 3752–3757 (2017).

[7]A. Cordaro, B. Edwards, V. Nikkhah, A. Alù, N. Engheta, and A. Polman, "Solving integral equations in free space with inverse-designed ultrathin optical metagratings," Nature Nanotechnology , 1–8 (2023).

[8]S.-H. Nam, M. Kim, N. Kim, D. Cho, M. Choi, J. H. Park, J. Shin, and S. Jeon, "Photolithographic realization of target nanostructures in 3d space by inverse design of phase modulation," Science Advances **8**, eabm6310 (2022).

[9]S. M. Kamali, E. Arbabi, H. Kwon, and A. Faraon, "Metasurface-generated complex 3-dimensional optical fields for interference lithography," Proceedings of the National Academy of Sciences **116**, 21379–21384 (2019).

[10]M. Schevenels, B. S. Lazarov, and O. Sigmund, "Robust topology optimization accounting for spatially varying manufacturing errors," Computer Methods in Applied Mechanics and Engineering **200**, 3613–3627 (2011).
